# Nurse-led group consultation intervention reduces depressive symptoms in men with localised prostate cancer: a cluster randomised controlled trial

**DOI:** 10.1186/s12885-016-2687-1

**Published:** 2016-08-16

**Authors:** Penelope Schofield, Karla Gough, Kerryann Lotfi-Jam, Rebecca Bergin, Anna Ugalde, Paul Dudgeon, Wallace Crellin, Kathryn Schubach, Farshard Foroudi, Keen Hun Tai, Gillian Duchesne, Rob Sanson-Fisher, Sanchia Aranda

**Affiliations:** 1 Department of Psychology, Swinburne University of Technology, Hawthorn, Australia; 2 Department of Cancer Experiences Research, Peter MacCallum Cancer Centre, 2 St Andrews Place, East Melbourne, Australia; 3 Sir Peter MacCallum Department of Oncology, Faculty of Medicine, Dentistry and Health Sciences, The University of Melbourne, Parkville, Australia; 4 School of Health Sciences, Faculty of Medicine, Dentistry and Health Sciences, The University of Melbourne, Parkville, Australia; 5 Deakin University, Faculty of Health, 221 Burwood Highway, Burwood, Australia; 6 Cancer Council Victoria, Cancer Information and Support Services, 615 St Kilda Rd, Melbourne, Australia; 7 School of Behavioural Science, University of Melbourne, Parkville, Australia; 8 Radiation Oncology and Cancer Imaging, Peter MacCallum Cancer Centre, 2 St Andrews Place, East Melbourne, Australia; 9 School of Medicine and Public Health, The University of Newcastle, University Drive, Callaghan, Australia; 10 Cancer Council Australia, Sydney, NSW Australia

**Keywords:** Prostate cancer, Radiotherapy, Intervention, Unmet needs, Psychological morbidity, Quality of life, Uro-oncology nurses

## Abstract

**Background:**

Radiotherapy for localised prostate cancer has many known and distressing side effects. The efficacy of group interventions for reducing psychological morbidity is lacking. This study investigated the relative benefits of a group nurse-led intervention on psychological morbidity, unmet needs, treatment-related concerns and prostate cancer-specific quality of life in men receiving curative intent radiotherapy for prostate cancer.

**Methods:**

This phase III, two-arm cluster randomised controlled trial included 331 men (consent rate: 72 %; attrition: 5 %) randomised to the intervention (*n* = 166) or usual care (*n* = 165). The intervention comprised four group and one individual consultation all delivered by specialist uro-oncology nurses. Primary outcomes were anxious and depressive symptoms as assessed by the Hospital Anxiety and Depression Scale. Unmet needs were assessed with the Supportive Care Needs Survey-SF34 Revised, treatment-related concerns with the Cancer Treatment Scale and quality of life with the Expanded Prostate Cancer Index −26. Assessments occurred before, at the end of and 6 months post-radiotherapy. Primary outcome analysis was by intention-to-treat and performed by fitting a linear mixed model to each outcome separately using all observed data.

**Results:**

Mixed models analysis indicated that group consultations had a significant beneficial effect on one of two primary endpoints, depressive symptoms (*p* = 0.009), and one of twelve secondary endpoints, procedural concerns related to cancer treatment (*p* = 0.049). Group consultations did not have a significant beneficial effect on generalised anxiety, unmet needs and prostate cancer-specific quality of life.

**Conclusions:**

Compared with individual consultations offered as part of usual care, the intervention provides a means of delivering patient education and is associated with modest reductions in depressive symptoms and procedural concerns. Future work should seek to confirm the clinical feasibility and cost-effectiveness of group interventions.

**Trial registration:**

Australian and New Zealand Clinical Trials Registry ANZCTRN012606000184572. 1 March 2006.

## Background

Radiotherapy is a commonly prescribed curative treatment for localised prostate cancer. Radiotherapy, however, has many known and distressing side effects including bowel and urinary urgency or incontinence and erectile dysfunction; these may persist many years post-treatment [1]. Enduring side effects can result in unmet needs [2], poorer health-related quality of life (HRQOL) [3] and ongoing psychological maladjustment [4–6]. Notably, such difficulties are pronounced in those receiving androgen deprivation therapy [1, 7].

The need for evidence-based interventions is clear, especially given the prevalence of prostate cancer and the often favourable long-term prognosis associated with localised disease [8]. Extant supportive care trials suggest that group-based interventions, involving one health professional and a group of patients, may provide an efficient and effective mode of delivering disease, treatment and self-management information [9]. More intensive, group-based interventions may also provide HRQOL and benefit finding advantages over basic information provision [10–12]. Specific evidence of efficacy for ameliorating psychological morbidity is lacking, but, to date, the impact of group-based interventions tailored to the *expressed needs* of group participants has not been evaluated. Further, previous trials have not targeted men commencing treatment at the *same* time.

The phase III randomised controlled trial (RCT) reported in this article assessed the relative benefits of a tailored, group consultation intervention for men receiving curative intent radiotherapy for prostate cancer compared with current best practice supportive care (or usual care) alone. Group consultations aimed to communicate information about diagnosis, treatment and side effects along with coaching in self-management. Group consultation content and discussions were tailored based on expressed needs and concerns. The primary hypothesis was that the group consultations would have a significant beneficial effect on psychological morbidity (anxious and depressive symptoms) compared with usual care alone. It was also hypothesised that the group consultations would have a significant beneficial effect on treatment-related concerns, unmet needs and prostate cancer-specific HRQOL.

## Methods

### Design

**Fig. 1:**
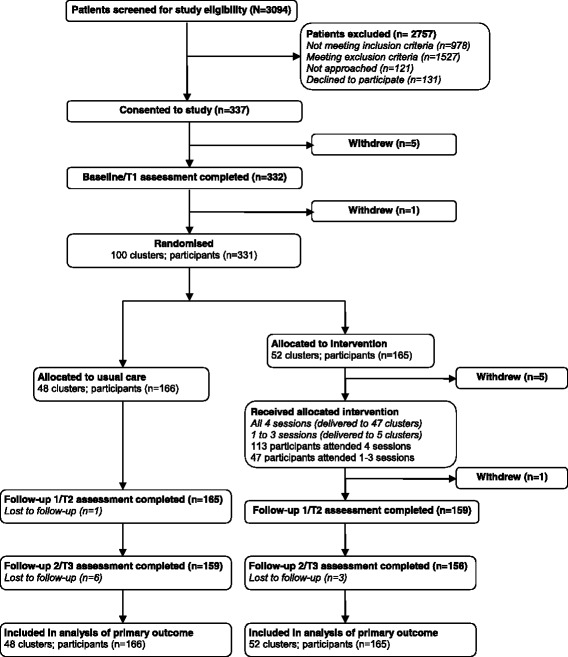
Participant flow following CONSORT guidelines

A two-arm, cluster RCT was used, intervention arm (*n* = 165) and control arm (*n* = 166); key components and timing are shown in Fig. 1. The unit of randomisation was all consenting patients scheduled to commence curative intent external beam radiotherapy for prostate cancer at two treatment sites within defined, consecutive fortnights. This ensured clusters allocated to the intervention arm would contain sufficient numbers of men to form a group and groups comprised men at similar stages in their treatment trajectory. Clusters were randomised remotely to the intervention or current best practice by a weighted-biased coin method. Neither participants nor statisticians were blinded. Participants were not blinded, because this is impossible in supportive care trials. Statisticians were unblinded after all trial outcome data was collected, but before preparation of the CONSORT flow diagram and outcome analyses. Randomisation was stratified by treatment site. Assessments occurred pre-treatment (T1), at the end of treatment (T2) and 6 months post-treatment (T3).

### Setting

This study was conducted at two sites of a specialist oncology facility, Peter MacCallum Cancer Centre, in Australia.

### Sample

Eligibility criteria required a confirmed diagnosis of prostate cancer; 18 years or older; commencing radical external beam radiotherapy with curative intent (with or without brachytherapy); and able to understand English. Patients with a serious cognitive or psychological disorder; who were scheduled to received palliative radiotherapy; having brachytherapy alone, had previous radiotherapy treatment; or deemed too unwell by the treatment team were excluded.

### Usual care: best practice supportive care

A nurse-led clinic forms part of usual care. All prostate cancer patients attend a minimum of one individual consultation with a specialist uro-oncology or radiation oncology nurse, then as required throughout their treatment. Nurses provide written information about treatment and side effects; referrals may be initiated also.

### Intervention: group consultations

Development and content of the group consultation intervention has been described in detail previously [13], so only a brief description follows.

The intervention package was designed to: 1) systematically assess patient needs and values to direct the content of consultations; 2) provide timely information on basic prostate anatomy, side effects, treatment and survivorship issues at critical points in the treatment trajectory; 3) coach men in evidence-based self-care and communication strategies with their treatment team to assist them to achieve optimal health status; and 4) offer a forum for psychosocial peer support and information exchange. It consists of four group consultations and one individual consultation.

All consultations comprising the intervention package were delivered by a specialist uro-oncology nurse. Group consultations were scheduled at critical times in the illness/treatment trajectory when patients often experience increased information needs and distress – specifically, beginning of treatment (week 1), mid-treatment (week 4), treatment completion (week 7) and 6-weeks post-treatment (week 13). The individual consultation was scheduled after the *beginning of treatment* group consultation. Note, also, that men could attend additional individual consultations after the *mid*-*treatment* and *treatment completion* group consultations as required.

The *beginning of treatment* group consultation focused on preparing men for radiotherapy treatment. The *mid*-*treatment* group consultation focused on educating men about common treatment side-effects and relevant self-care strategies and normalising the impact of these side effects. The *treatment completion* consultation reinforced and elaborated on the content and discussions of the *mid*-*treatment* session to maximise the use of self-care and communication strategies. The *treatment completion* consultation also focused on helping men achieve a sense of closure following treatment and manage any concerns the may have had for the future (e.g., returning to work). The *6*-*weeks post*-*treatment* consultation dealt with possible late sexual side effects of radiotherapy treatment and cancer survivorship issues including fear of cancer recurrence.

Parts of the *beginning of treatment* consultation and most of the *mid*-*treatment* consultation were tailored to the needs of group participants based on their responses to two separate question prompt lists. Men’s responses to the question prompt list administered at the *beginning of treatment* group consultation were also used to guide their individual consultation with the intervention nurse.

#### Application of intervention protocol

Group-based consultations of approximately one hour were run by one of three specialist uro-oncology nurses trained in group facilitation skills and the intervention package. An intervention manual, summarising details of the intervention, was developed to support nurse training. Specialist uro-oncology nurses had minimal interaction with men allocated to usual care to reduce ‘contamination’ between arms. If intervention patients were unable to attend in person, they joined the group consultation via telephone or received a catch-up session.

#### Quality assurance

36 of 193 tape-recorded consultation sessions were randomly selected and assessed for adherence to the intervention protocol by an independent rater against a checklist of intervention elements. On average, 74 % of the intervention manual content was delivered, consistent with the tailored nature of the material.

### Recruitment and assessment procedures

A trained research assistant identified and approached potentially eligible participants from outpatient clinic and treatment lists between 2nd January 2007 and 18th December 2009. Written informed consent and baseline self-report questionnaires were completed prior to randomisation. Follow-up questionnaires were completed at hospital appointments or at home and returned via post.

### Measures

Demographic and clinical information for consenters and decliners was gathered from medical records. Reasons for refusal were recorded.

Psychological morbidity and global distress were assessed with the Hospital Anxiety and Depression Scale (HADS). The 14-item HADS comprises two subscales designed to assess anxious (HADS-A) and depressive (HADS-D) symptomatology in the past week [14]. The single-item DT provides a measure of global distress experienced in the past 7 days [15]. Cancer treatment-related concerns were measured with the Cancer Treatment Scale (CaTS) [16]. The 25-item CaTS comprises two subscales assessing patients’ sensory/psychological and procedural concerns about their upcoming treatment. Unmet supportive care needs were assessed with the Supportive Care Needs Survey short-form revised (SCNS-SF34-R). The 34-item SCNS-SF34-R comprises five subscales measuring levels of unmet psychological, health system and information, physical and daily living, patient care and support and sexuality needs in the last month [17]. Prostate cancer-specific HRQOL was assessed with the Expanded Prostate Cancer Index Composite short-form (EPIC-26) [18]. The 26-item EPIC-26 comprises four subscales examining functioning and symptom bother relevant to the urinary, bowel, hormonal and sexual domains.

### Power considerations

Using methods proposed by Eldridge et al. [19], the estimated design effect for the study was 1.17 (based on 100 clusters of average size 4, a coefficient of variation for cluster size of 0.25 and a conservative at-worst intra-class correlation of 0.05) [20]. Initial sample size calculations incorporated this estimated design effect and were calculated for feasible treatment effect differences of 0.35 SD for continuous outcomes. To achieve at least 80 % power at a 5 % significance level, 130 × 1.17 = 152 patients in each arm were required.

### Statistical analysis

Pearson’s χ^2^ or Fisher’s exact test for nominal variables, Mann–Whitney *U*-tests for ordinal variables and independent samples *t*-tests for continuous variables were used to compare characteristics of study participants and study decliners. Descriptive statistics were used to examine questionnaire compliance and summarise patient characteristics and responses to outcomes measures by study arm at baseline and follow-up assessments.

Primary outcome analysis was by intention-to-treat and performed by fitting a linear mixed model to each outcome separately using all observed data. Missing data imputation was not undertaken. Three-level models (Level 1, time point; Level 2, participant; and Level 3, cluster) including random intercepts and slopes were constructed for each outcome following recommended procedures for multi-level modelling [21]. Fully parameterised models also included fixed effects for time (linear and quadratic components: time and quadtime), group (usual care, intervention), site (1, 2), pre-baseline androgen deprivation therapy (pre-BL ADT: yes, no) plus two-way and cross-level interactions. Pre-BL ADT was included as a covariate as previous research indicates a robust relationship between hormone therapy and study outcomes [1, 7], but parameters were retained only if normal distribution tests were significant and the more elaborate models provide a better fit to the data. Random slope terms were also only retained if there was significant variance between participants.

As a secondary descriptive analysis, individual change scores were calculated between T1 and follow-ups at T2 and T3. The within-group effect size was calculated as the mean change from baseline divided by the standard deviation at baseline. The between-group effect size was calculated as the difference between study arms in mean change from baseline divided by the pooled standard deviation of change [22].

All exploratory and descriptive analyses were performed with SPSS Windows Version 18.0 (SPSS, Chicago, IL, USA). Outcome analyses were performed with MLwiN Version 2.1 [23].

## Results

### Trial profile

Of 589 patients who were eligible for the study (Fig. 1), 468 patients were approached and 337 consented to participate (72 % consent rate). Of the 331 patients randomised, 166 were allocated to usual care via 48 clusters (median size 2 patients, IQR 2–4) and 165 to the intervention via 52 clusters (median size 3 patients, IQR 2–4.75).

Apart from a higher consent rate at one site (*p* = 0.02), none of the associations between patient characteristics and response status or group differences between consenters and decliners were statistically significant (Table 1). Study arms appeared well balanced in terms of baseline characteristics (Table 1).

**Table 1:** Patient demographic and clinical characteristics of consenters (by study arm) and decliners

	Consenters						*P*
	Usual care		Intervention		Decliners		
	(*N* = 166)		(*N* = 165)		(*N* = 115)		
	*n*	%	*n*	%	*n*	%	
Oncology facility							0.02
Site 1	70	42.2	70	42.4	64	55.7	
Site 2	96	57.8	95	57.6	51	44.3	
Age at baseline, years							0.30
Mean	67.6		67.2		68.1		
Standard Deviation	6.7		6.9		7.9		
Range	46–85		51–84		44–82		
<65	54	32.5	56	33.9	32	27.8	
≥65	112	67.5	109	66.1	83	72.2	
Marital status							
Married/defacto	136	81.9	127	77.0			
Other	30	18.1	38	23.0			
Location							0.14
Urban	136	81.9	136	82.4	101	87.8	
Rural	30	18.1	29	17.6	13	11.3	
Missing					1		
Risk group (D’Amico, 1998)							0.16
Low	11	10.6	11	11.6	4	3.9	
Intermediate	44	42.3	39	41.1	37	35.9	
High	49	47.1	45	47.4	38	36.9	
Scheduled treatment							0.29
Salvage EBRT	61	36.7	69	41.8	31	27.0	
Brachytherapy followed by EBRT	8	4.8	9	5.5	8	7.0	
EBRT followed by brachytherapy	5	3.0	3	1.8	1	0.9	
EBRT alone	92	55.4	84	50.9	70	60.9	
Previous treatment							
Any previous treatment							0.60
No	39	23.5	34	20.6	28	24.3	
Yes	127	76.5	131	79.4	87	75.7	
Active surveillance							1.00
No	148	89.2	153	92.7	105	91.3	
Yes	18	10.8	12	7.3	10	8.7	
Prostatectomy							0.15
No	105	63.3	96	58.2	79	68.7	
Yes	61	36.7	69	41.8	36	31.3	
Androgen deprivation							0.49
No	113	68.1	116	70.3	75	65.2	
Yes	53	31.9	49	29.7	40	34.8	

*P*-value relates to comparison of consenters versus decliners. Other marital status includes never married, separated/divorced and widowed. Risk groups were designated for men who did not undergo surgery

*EBRT* external beam radiotherapy, *TURP* transurethral resection of the prostate

### Intervention fidelity

All four group consultation sessions were delivered to 47 intervention clusters; 1–3 sessions were delivered to the remaining 5 clusters. In total, 113 participants attended all group consultations, 34 attended three, 10 attended two, 3 one, and 5 none. The reasons for missed consultations were: scheduling issues, distance from hospital, study withdrawal, patient too unwell or no reason given. Of the 52 men who missed at least one session, 18 attended a catch-up consultation.

### Questionnaire compliance

Questionnaire compliance was high: > 96 of participants provided data on all outcomes at T1, > 95 at T2 and > 92 % at T3 (available on request from the authors).

### Outcome analyses

Descriptives for outcome measures by study arm at baseline and follow-up assessments are provided in Table 2. Results from the mixed models and secondary descriptive analyses are provided in Tables 3 and 4 respectively (estimates of variance components and cluster level ICC are available on request from the authors).

**Table 2:** Descriptives for study measures by study arm at baseline and follow-up assessments

	Assessment					
	Baseline/before radiotherapy		End of radiotherapy		6 months post-radiotherapy	
Outcome by study arm	M	SD	M	SD	M	SD
Hospital Anxiety and Depression Scale						
Anxiety						
Usual care	4.5	(3.5)	3.8	(3.2)	3.9	(3.3)
Intervention	4.4	(3.5)	3.4	(3.1)	3.8	(3.8)
Depression						
Usual care	2.6	(2.8)	3.1	(2.9)	3.0	(3.2)
Intervention	2.8	(2.9)	2.6	(2.4)	2.9	(2.9)
Distress Thermometer						
Usual care	2.0	(2.2)	2.1	(2.3)	1.7	(2.1)
Intervention	1.9	(2.3)	1.7	(2.3)	1.5	(2.3)
Expanded Prostate cancer Index Composite						
Bowel						
Usual care	92.9	(12.4)	77.6	(20.1)	86.5	(16.8)
Intervention	93.5	(12.7)	80.6	(18.2)	87.6	(14.7)
Urinary						
Usual care	84.5	(16.1)	73.6	(17.9)	83.5	(16.8)
Intervention	85.2	(14.9)	76.0	(17.5)	83.0	(16.0)
Sexual						
Usual care	31.8	(28.7)	26.2	(25.4)	27.2	(26.7)
Intervention	27.0	(25.8)	23.8	(23.1)	25.0	(24.6)
Hormonal						
Usual care	84.3	(18.3)	82.5	(17.3)	83.4	(18.6)
Intervention	82.9	(19.1)	83.1	(18.5)	84.4	(19.8)
Supportive Care Needs Survey						
Psychological						
Usual care	23.9	(21.4)	19.6	(21.2)	15.6	(21.0)
Intervention	22.7	(21.4)	18.4	(20.4)	14.7	(20.0)
Health system & information						
Usual care	28.4	(30.2)	21.4	(24.2)	17.0	(23.7)
Intervention	27.1	(29.4)	19.3	(25.6)	13.2	(21.1)
Patient care & support						
Usual care	12.1	(18.2)	12.2	(18.6)	8.3	(16.0)
Intervention	11.1	(17.9)	9.9	(16.1)	9.0	(19.2)
Physical & daily living						
Usual care	13.7	(22.1)	19.6	(21.2)	15.6	(21.0)
Intervention	11.5	(18.1)	18.4	(20.4)	14.7	(20.0)
Sexual						
Usual care	26.5	(29.4)	22.9	(28.3)	27.2	(30.8)
Intervention	27.2	(29.6)	22.4	(27.7)	26.1	(28.3)
Cancer Treatment Survey						
Procedural						
Usual care	2.7	(1.0)	1.9	(.9)		
Intervention	2.7	(1.1)	1.8	(.9)		
Sensory/psychological						
Usual care	2.3	(.9)	1.7	(.7)		
Intervention	2.3	(.9)	1.6	(.8)		

For the Hospital Anxiety and Depression Scale, higher scores reflect higher levels of anxious and depressive symptomatology. For the Distress Thermometer, higher scores reflect higher levels of distress. For the Expanded Prostate cancer Index Composite-26, higher scores reflect higher quality of life/better functioning/lower bother. For the Supportive Care Needs Survey (Short Form with Revised response scale), higher scores reflect higher levels of unmet need. For the Cancer Treatment Scale, higher scores reflect higher levels of cancer treatment-related concerns

**Table 3:** Mixed models results for primary and secondary outcomes: estimates of fixed effects

Parameter	HADS						DT			EPIC-26											
	Anxiety			Depression						Bowel			Urinary			Sexual			Hormonal		
	est		s.e.	est		s.e.	est		s.e.	est		s.e.	est		s.e.	est		s.e.	est		s.e.
Intercept	3.9	*	0.4	1.5	**	0.3	1.5	**	0.3	94.5	**	1.9	85.0	**	2.0	38.6	**	3.1	92.2	**	1.9
Time	−1.3	**	0.4	0.8	**	0.3	−0.2		0.3	−24.7	**	2.5	−14.2	**	2.1	−4.4		2.3	0.1		1.8
Quadtime	0.3	**	0.1	−0.2	*	0.1	0.0		0.1	5.9	**	0.6	3.6	**	0.5	0.8		0.5	−0.1		0.4
Group	0.6		0.5	1.0	*	0.5	0.3		0.3	1.0		2.6	0.2		2.6	−5.7		4.2	−5		2.6
Site	1.1	*	0.6	1.1	*	0.5	0.7		0.3	−2.2		2.3	−1.1		2.4	−2.4		3.9	−2.2		2.4
Pre-BL ADT	1.3	*	0.5	2.2	**	0.5	0.8	**	0.3	−0.9		2.4	0.3		2.0	−16.6	**	2.9	−20.4	**	1.6
Group × Site	−1.4	*	0.7	−1.4	*	0.6	−0.8		0.4	1.7		2.8	1.0		3.2	1.3		5.2	4.8		3.2
Group × Pre-BL ADT										−5.2		3.0									
Site × Pre-BL ADT	−2.3	**	0.7	−1.6	**	0.6	−1.0		0.4												
Group × Time	−0.3		0.4	−1.0	**	0.3	−0.5		0.3	3.6		2.5	2.2		2.1	2.7		2.4	2.3		1.9
Group × Quadtime	0.1		0.1	0.2	**	0.1	0.1		0.1	−0.9		0.6	−0.7		0.5	−0.5		0.6	−0.4		0.5
Time × Site	0.5		0.4	0.4		0.3	0.6		0.3	5.6	*	2.5	3.5		2.1	−4.2		2.4	−4.2	*	2.0
Quadtime × Site	−0.1		0.1	−0.1		0.1	−0.1		0.1	−1.5	*	0.6	−0.9		0.5	0.9		0.6	1.0	*	0.5
Time × Pre-BL ADT	0.2	**	0.1	−0.9	**	0.3				4.2		2.7	−6.9	**	2.3	1.2	*	0.6			
Quadtime × Pre-BL ADT				0.2	*	0.1				−1.2		0.6	1.5	**	0.5						
Parameter	SCNS-SF34-R															CaTS					
	Psychological			Health system & information			Patient care & support			Physical & daily living			Sexuality			Procedural			Sensory/ psychological		
Intercept	18.8	**	2.5	26.4	**	3.0	8.7	**	2.0	6.4	**	2.4	20.3	**	3.3	2.6	**	0.1	2.1	**	0.1
Time	−7.2	**	2.3	−9.0	**	3.5	0.3		2.4	8.2	**	2.5	−5.2		3.4	−0.8	**	0.1	−0.6	**	0.1
Quadtime	1.2	*	0.5	1.4		0.8	−0.4		0.6	−1.9	**	0.6	1.1		0.8						
Group	4.2		3.2	0.3		3.9	1.3		2.7	1.7		3.1	5.4		4.4	0.0		0.2	0.1		0.1
Site	7.1	*	3.2	−0.3		3.7	3.4		2.5	7.5	*	2.9	6.7		4.1	0.0		0.1	0.2		0.1
Pre-BL ADT	9.0	**	3.0	6.7	**	2.5	4.4	**	1.7	9.3	**	2.0	7.4	*	2.9	0.2	*	0.1	0.2	**	0.1
Group × Site	−10.0	**	3.8	−2.4		4.7	−3.9		3.2	−6.3		3.8	−7.8		5.4	0.0		0.2	−0.1		0.2
Site × Pre-BL ADT	−9.0	*	4.0																		
Group × Time	−3.2		2.5	−0.9		3.8	−1.7		2.7	1.0		2.7	−1.5		3.7	−0.2	*	0.1	−0.1		0.1
Group × Quadtime	0.7		0.6	0.1		0.9	0.6		0.6	−0.1		0.6	0.3		0.9						
Time × Site	3.9		2.5	1.3		3.9	0.4		2.7	−0.6		2.7	0.4		3.7	0.2		0.1	0.1		0.1
Quadtime × Site	−0.7		0.6	0.0		0.9	0.1		0.6	0.1		0.6	0.2		0.9						

The coefficient for each group by time interaction represents the average difference in rate of change for the intervention group relative to the usual care group. The coefficient for each group by quadtime interaction represents the average difference in “change” in the rate of change (acceleration or deceleration) for the intervention group relative to the usual care group

Time represents average number of months since first assessment. Reference categories: group, usual care; site, Site 2; and pre-BL ADT, no. Time modelled as a random effect for HADS Anxiety and Depression, EPIC-26 Hormonal and Sexual Summary and SCNS-SF34-R Psychological and Sexuality. Terms for the interaction between Pre-BL ADT and Group, Time and Quadtime were not included in the final models for SCNS-SF34-R and CaTS subscales. In all other cases where the estimate of a coefficient is not provided, the relevant term was not included in the final model. * *p* < .05; ** *p* < .01

Variance components are available from the authors

**Table 4:** Mean change from baseline and effect size at the end of radiotherapy and 6 months post-radiotherapy

		Usual care			Intervention			Between-groups difference	95 % CI	Effect size
		Mean change from baseline	95 % CI	Effect size	Mean change from baseline	95 % CI	Effect size			
End of radiotherapy										
HADS	Anxiety	−0.7	−1.2, −0.3	0.21	−1.0	−1.4, −0.6	0.29	−0.2	−0.8, 0.4	0.09
	Depression	0.6	0.3, 0.9	0.21	−0.2	−0.6, 0.1	0.07	−0.8	−1.2, −0.3	0.37
DT		0.1	−0.3, 0.5	0.05	−0.3	−0.7, 0.2	0.12	−0.4	−0.9, 0.2	0.15
EPIC-26	Urinary	−11.0	−13.4, −8.6	0.68	−9.2	−11.5, −6.9	0.62	1.8	−1.5, 5.1	0.12
	Bowel	−15.7	−18.4, −12.9	1.34	−12.8	−15.8, −9.8	0.99	2.9	−1.1, 6.9	0.16
	Sexual	−5.2	−7.9, −2.5	0.18	−2.8	−5.5, −0.2	0.11	2.4	−1.4, 6.1	0.14
	Hormonal	−1.8	−4.0, 0.3	0.10	0.0	−2.2, 2.2	0.00	1.9	−1.2, 4.9	0.14
SCNS-SF34-R	Physical & daily living	6.0	3.0, 9.1	0.27	6.8	4.0, 9.7	0.37	0.8	−3.3, 5.0	0.04
	Psychological	−4.1	−6.9, −1.3	0.20	−6.7	−9.4, −4.0	0.32	−2.6	−6.5, 1.3	0.15
	Sexuality	−3.8	−7.8, 0.2	0.13	−4.9	−9.3, −0.5	0.17	−1.1	−7.0, 4.8	0.04
	Patient care and support	0.2	−2.5, 2.9	0.01	−0.8	−3.9, 2.3	0.05	−1.0	−5.1, 3.1	0.05
	Health system & information	−6.9	−11.0, −2.9	0.23	−7.6	−12.4, −2.8	0.26	−0.7	−6.9, 5.6	0.02
CaTS	Sensory/psychological	−0.6	−0.7, −0.5	0.64	−0.7	−0.8, −0.5	0.72	−0.1	−0.3, 0.1	0.10
	Procedural	−0.7	−0.9, −0.6	0.71	−1.0	−1.1, −0.8	0.86	−0.2	−0.5, −0.02	0.24
6 months post-radiotherapy										
HADS	Anxiety	−0.6	−1.1, −0.2	0.18	−0.6	−1.1, −0.1	0.18	0.0	−0.7, 0.7	0.01
	Depression	0.4	0.04, 0.8	0.16	0.1	−0.3, 0.5	0.04	−0.3	−0.9, 0.2	0.14
DT		−0.3	−0.6, 0.04	0.14	−0.5	−0.8, −0.2	0.21	−0.2	−0.7, 0.3	0.10
EPIC-26	Urinary	−1.0	−2.9, 0.9	0.06	−2.2	−4.5, 0.1	0.15	−1.2	−4.2, 1.8	0.09
	Bowel	−6.8	−9.4, −4.3	0.55	−6.2	−8.5, −3.9	0.57	0.6	−2.8, 4.1	0.04
	Sexual	−4.4	−8.0, −0.9	0.15	−2.1	−5.1, 0.9	0.08	2.3	−2.4, 7.1	0.11
	Hormonal	−0.8	−3.2, 1.6	0.05	1.3	−1.3, 4.0	0.07	2.2	−1.4, 5.7	0.14
SCNS-SF34-R	Physical & daily living	1.7	−1.4, 4.8	0.07	3.4	0.7, 6.1	0.19	1.8	−2.3, 5.9	0.10
	Psychological	−7.2	−10.2, −4.3	0.35	−8.0	−10.8, −5.3	0.39	−0.8	−4.9, 3.3	0.04
	Sexuality	0.0	−4.6, 4.5	0.00	−1.2	−5.5, 3.1	0.04	−1.2	−7.4, 5.1	0.04
	Patient care and support	−3.5	−6.1, −1.0	0.20	−1.7	−4.9, 1.5	0.10	1.8	−2.3, 5.9	0.10
	Health system & information	−10.9	−14.9, −7.0	0.37	−13.4	−17.8, −9.0	0.46	−2.5	−8.4, 3.5	0.09

Effect sizes for changes from baseline = (Mean change from baseline/standard deviation at baseline)

Effect sizes for between-groups differences = ((Intervention Mean change from baseline) – (Usual Care Mean change from baseline)) / pooled standard deviation for change. For HADS, DT, SCNS-SF34-R and CaTS, a score decrease reflects improvement; as such, between-groups differences with a negative sign indicate a greater improvement (or lesser deterioration) among intervention participants. For EPIC-26, a score increase reflects improvement; as such, between-groups differences with a positive sign indicate a greater improvement (or lesser deterioration) among intervention participants

### Primary outcomes

#### Depressive symptoms

For HADS-D, apart from the site by time interactions, all fixed effects were statistically significant (all *p* > 0.05; Table 3). The difference in the rate of change on the HADS-D for the intervention group relative to the usual care group was significant (*p* = 0.0009). Change in the rate of change was also significant (*p* = 0.001). Irrespective of group, patients who had received pre-BL ADT had higher levels of depressive symptoms at baseline (*p* < 0.0001) and exhibited greater reductions in these symptoms at T2 and T3 compared with those who had not received ADT (*p* = 0.008).

Descriptive analysis indicated a slight reduction in depressive symptoms in the intervention group between baseline and end of RT, whereas the usual care group reported an increase in these symptoms in the same time period (*M* chg = −0.2 and 0.6 respectively; *M* diff = −0.8, 95 % CI: −1.2, −0.3, Table 4). The effect size for the between-groups difference at the end of radiotherapy was 0.37. The difference between groups persisted 6 months post-RT, although the between-groups difference in mean changes was substantially reduced (*M* diff = −0.3, 95 % CI: −0.9, 0.2; effect size = 0.14).

#### Anxious symptoms

For HADS-A, the difference in rate of change for the intervention group relative to the usual care group was not significant (*p* = 0.42, Table 3). Irrespective of group, pre-BL ADT patients exhibited a lower rate of decline in anxiety per follow-up compared with those who had not received pre-BL ADT (*p* = 0.008).

Descriptive analysis indicated a reduction in anxious symptoms for both groups at follow-up assessments from baseline levels (Table 2). However, consistent with the mixed models results, differences in mean changes from baseline at the end of radiotherapy (*M* diff = −0.2, 95 % CI: −0.8, 0.4; effect size = 0.09) and 6 months post-radiotherapy (*M* diff = 0.0, 95 % CI: −0.7, 0.7; effect size = 0.01) were negligible.

### Secondary outcomes

#### Global distress

For the DT, the difference in rate of change for the intervention group relative to the usual care group was not significant (*p* = 0.16, Table 3). Irrespective of group, pre-BL ADT patients reported higher levels of global distress at baseline (*p* = 0.008). The effect sizes for between-groups differences in mean changes at both follow-ups were 0.15 and 0.1 respectively (Table 4).

#### Prostate cancer-specific HRQoL

For EPIC-26 domains, none of the differences in rate of change for the intervention group relative to the usual care group were significant (all *p* > 0.05, Table 3). Notably, however, for the Bowel and Urinary summaries, model coefficients for time and quadtime were highly significant (all *p* < 0.001) and pre-BL ADT patients exhibited a significantly greater decline in urinary scores per follow-up compared with those who had not received pre-BL ADT (*p* < 0.0025).

For both usual care and intervention groups, descriptive analysis indicated medium- to large-sized deterioration in urinary (effect size for within-group changes = 0.68 and 0.62, respectively) and bowel functioning (effect size for within-group changes = 1.34 and 0.99, respectively) at the end of RT compared to baseline levels (Table 4). Six months post-RT, however, urinary functioning was comparable with baseline levels for both groups (effect size for within-group changes = 0.06 and 0.15, respectively), whereas bowel functioning was still somewhat worse (effect size for within-group changes = 0.55 and 0.57 respectively). Effect sizes for all between-groups differences in mean changes at both follow-ups were trivial- to small-sized (range = 0.04 to 0.16; Table 4).

#### Unmet supportive care needs

For SCNS-SF34-R domains, none of the differences in rate of change for intervention relative to usual care were significant (all *p* > 0.05, Table 3). Notably, baseline levels of patient care and support and physical and daily living needs were very low (i.e., estimates for Intercept, Table 3). Effect sizes for all between-groups differences in mean changes at both follow-ups were trivial- to small-sized (range = 0.02 to 0.15; Table 4).

#### Cancer treatment-related concerns

There was a significant reduction in both types of cancer treatment-related concerns for both groups between baseline and end of RT (both *p* < 0.001; Table 3). Relative to the usual care group, there was a statistically significant reduction in procedural concerns for the intervention group (*p* = 0.049), however no intervention benefit was observed for sensory/psychological concerns (*p* = 0.46). The effect size for the between-groups difference for procedural concerns was 0.24 (Table 4).

## Discussion

This study assessed the relative benefits of a tailored, group consultation intervention for men receiving curative intent radiotherapy for prostate cancer compared with current best practice supportive care alone. A key innovation was the requirement that group content and discussions be tailored based on men’s expressed needs and concerns. Information provided was high-quality, evidence-based and appropriately timed. Discussions provided opportunities for peer support, including emotional and practical sharing, and men often saw each other in the treatment clinic waiting rooms, reinforcing their shared experience. Intervention fidelity was moderate: all four group consultations were delivered to 47 of 52 (90 %) clusters and a majority of intervention participants attended all four group consultations (113 of 165) or a catch-up consultation (18 of 52).

A modest intervention benefit was demonstrated on one of two primary outcomes, depressive symptoms, and one of twelve secondary outcomes, treatment-related procedural concerns. The intervention benefit for depressive symptoms was most evident at the end of radiotherapy; compared to baseline, intervention participants reported a slight reduction in depressive symptoms, whereas usual care participants reported an increase in these symptoms. The benefit for treatment-related procedural concerns was also observed at the end of radiotherapy. Intervention benefits as assessed by all other study outcomes were trivial- to small-sized and non-significant.

This is the first trial of a group-based intervention for prostate cancer patients to demonstrate a significant beneficial effect on depressive symptoms, as assessed by the HADS-D. Previous trials using depression as an outcome have shown little, if any, impact on depressive symptoms in the short- or long-term [24, 25]. Notably, items comprising the HADS-D concentrate on an inability to experience pleasure [26]. Evidence suggests anhedonia is more common than depressed mood among prostate cancer patients, possibly because of reduced sources of pleasure or reduced ability to access those sources [27]. Speculatively, participation in our group consultation intervention may have helped to normalise men’s experiences and bolster hope, offsetting the increase in depressive symptoms reported by usual care participants. Previously tested interventions either provided no or substantially fewer opportunities for peer support, which has been emphasised as a possible mechanism of effect in group-based interventions [9, 11, 25]. While the size of the benefit on depressive symptoms was modest (effect size = 0.37), it should be considered in the context of floor effects on the HADS-D and the medium-to-large-sized deterioration in prostate cancer-specific HRQOL following radiotherapy.

The intervention benefit on treatment-related procedural concerns is also noteworthy. Together with the results from our recent trial of a nurse-led pre-chemotherapy education intervention [28], the current findings suggest that well-structured and appropriately timed nurse-led consultations can be effective in reducing cancer treatment-related concerns, especially those related to the procedural aspects of treatment.

There was no evidence that group consultations afforded a prostate cancer-specific HRQOL advantage or provided benefits in terms of unmet needs. Previous trials have used general HRQOL as an outcome [10–12], rather than prostate cancer-specific HRQOL, making it impossible to compare the results. Nevertheless, recent evidence suggests that functioning and symptom bother related to prostate cancer and its treatment may require much more targeted and intensive intervention than that offered to either study arm in this trial [29].

Regarding limitations, the current trial was conducted in a specialist cancer centre with a high standard of usual care. Men randomised to usual care also received evidence-based information about upcoming treatment, likely side effects and self-care strategies; however the precise details of information provided in usual care were not assessed. Intervention effects, or the lack thereof, should be considered in this context. Further, with the exception of sexual functioning, baseline functioning was uniformly high. While not so at the time this trial was designed, it is now widely recognised that floor effects (and/or not preselecting trial participants based on the need for help) may inadvertently lead to an underestimation of intervention effects [30].

Traditionally, psycho-educational interventions have been formulaic and didactic with static content. They have comprised people with different cancers, people at different stages in the illness trajectory and people receiving different treatments. The current trial design ensured group consultations were composed of men at a very similar stage in the treatment trajectory: all commencing potentially curative treatment for prostate cancer, then receiving daily radiotherapy across approximately the same timeframe. Tailoring ensured the relevance of educational content and group discussions to all participants and had a significant beneficial effect on depressive symptoms and procedural concerns.

## Conclusions

HRQOL and unmet needs advantages were not observed, but, arguably, these findings suggest that group consultations provide an efficient and effective means of delivering patient education. Future work should seek to confirm the clinical feasibility of implementing this nurse-led group consultation, particularly amongst men who have depressive symptoms.
